# Estimation of cost-effectiveness in randomised controlled trials when not all participants allocated to active intervention receive it

**DOI:** 10.1186/1745-6215-12-S1-A45

**Published:** 2011-12-13

**Authors:** Chris Metcalfe

**Affiliations:** 1 MRC ConDuCT Hub for Trials Methodology Research, School for Social and Community Medicine, University of Bristol, Canynge Hall, 39 Whatley Road, Bristol, BS8 2PS, UK

## Objectives

In randomised controlled trials (RCTs) with some participants allocated to active intervention not complying with it, intention-to-treat analyses are recognised as underestimating the treatment effect on those participants who do comply. As compliance with treatment is often associated with prognosis, the alternative per protocol analysis will usually give a biased estimate of the treatment effect. Methods of unbiased estimation of the treatment effect in compliers have been developed for clinical outcome measures, but are not routinely employed in the estimation of cost effectiveness. A systematic review examines the accommodation of non-compliance in published RCT-based cost-effectiveness analyses.

## Methods

A systematic review of published RCTs is being undertaken, examining how patients who did not comply with allocated treatment were incorporated in estimates of cost-effectiveness. This abstract reports on preliminary work, searching for “cost-effectiveness” and “cost-utility” in the titles and abstracts of research articles published in the BMJ during 2009 and 2010.

## Results

Of 27 articles found, 11 were RCTs with a cost-effectiveness analysis. It was not clear from the reports of 4/11 trials whether any non-compliance occurred, with little detail given on the extent of non-compliance in a further 3 studies. In none of these seven studies was the effect of non-compliance on estimates of cost-effectiveness considered. Of the remaining studies, one conducted an intention-to-treat analysis for the clinical outcome but appeared to omit non-compliers from the estimates of cost; one study calculated intention-to-treat estimates of effect on the clinical and the cost outcomes and an unbiased estimate of the clinical effect in compliers; and one study included non-compliers in their allocated groups for the main analysis but conducted a sensitivity analysis of cost-effectiveness with these people excluded. The fourth was a cluster-randomised trial, which presented costs and clinical effects per catchment area and costs per individual receiving the intervention.

## Conclusions

Preliminary findings suggest that the majority of studies take an intention-to-treat approach to comparing costs between randomly allocated treatments. However, it is unclear whether the costs of allocated or of received treatment are contributing to these estimates. A minority of studies are conducting secondary per protocol analyses, with no study attempting an unbiased estimate of cost-effectiveness in compliers. How the costs of partial compliance, which may be associated with treatment success, can be incorporated into these estimates without causing bias is unclear.

